# Myocardial infarction due to thrombotic occlusion despite anticoagulation in Kawasaki disease – a case report

**DOI:** 10.1186/s12887-022-03151-2

**Published:** 2022-02-12

**Authors:** Diana van Stijn, Nikki J. Schoenmaker, R. Nils Planken, Dave R. Koolbergen, Samantha C. Gouw, Taco W. Kuijpers, Nico A. Blom, Irene M. Kuipers

**Affiliations:** 1 Department of Pediatric Immunology, Rheumatology and Infectious diseases, Emma Children’s Hospital, Amsterdam UMC, University of Amsterdam, Meibergdreef 9, location H8-253, Amsterdam, 1105 AZ The Netherlands; 2 Department Pediatric Intensive Care Unit (PICU), Emma Children’s Hospital, Amsterdam UMC, University of Amsterdam, Amsterdam, The Netherlands; 3 Department of Radiology and Nuclear medicine, Amsterdam UMC, University of Amsterdam, Amsterdam, The Netherlands; 4 Department of Cardiothoracic Surgery, Amsterdam UMC, University of Amsterdam, Amsterdam, The Netherlands; 5 Department of Pediatric Hematology, Emma Children’s Hospital, Amsterdam UMC, University of Amsterdam, Amsterdam, The Netherlands; 6 Department of Pediatric cardiology, Emma Children’s Hospital, Amsterdam UMC, University of Amsterdam, Amsterdam, The Netherlands

**Keywords:** Coronary artery aneurysms, Thrombosis, Clopidogrel resistance, Imaging, Antiplatelet and anticoagulant drugs

## Abstract

**Background:**

Kawasaki disease (KD) is a pediatric vasculitis. Mainly the coronary arteries become affected due to acute inflammation and formation of coronary artery aneurysms (CAAs) can occur. The larger the CAA, the higher the risk for clinical complications and major adverse cardiac events, as the blood flow changes to vortex or turbulent flow facilitating thrombosis. Such patients may develop life threatening thrombotic coronary artery occlusion and myocardial ischemiaunless anti-platelet and anti-coagulation therapy is timely initiated.

**Case presentation:**

We present a unique case of a 5-year-old girl with KD associated giant CAAs suffering from myocardial ischemia due to acute progressive thrombus growth despite intensive anticoagulation treatment (acetylsalicylic acid, acenocoumarol and clopidogrel) after 21 months of onset of disease. Thrombus growth continued even after percutaneous coronary intervention (PCI) with thrombolytic treatment and subsequent systemic thrombolysis, finally causing lasting myocardial damage. Acute coronary artery bypass grafting (CABG) was performed, although technically challenging at this very young age. Whereas myocardial infarction was not prevented, follow-up fortunately showed favorable recovery of heart failure.

**Conclusions:**

Anticoagulation and thrombolysis may be insufficient for treatment of acute coronary syndrome in case of impending thrombotic occlusion of giant coronary aneurysms in KD. Our case demonstrates that a thrombus can still continue to grow despite triple anticoagulation therapy and well-tailored cardiovascular follow-up, which can be most likely attributed to the state of low blood flow inside the aneurysm.

**Supplementary Information:**

The online version contains supplementary material available at 10.1186/s12887-022-03151-2.

## Background

Kawasaki disease (KD) is a systemic vasculitis that mainly affects children < 5 years of age [1]. KD was first described in 1967, however the etiology of KD is still unknown. Experience of the past 60 years demonstrated that KD patients are at risk for the development of coronary artery aneurysms (CAAs). Approximately 25% of untreated patients develop CAAs [2], and 4–16% if treated timely with intravenous immunoglobulin (IVIG) [1].

These aneurysms can be classified according to their *Z* score as “small-sized” (≥ 2,5 < 5.0), “medium-sized” (≥ 5.0 < 10.0) or “giant”(≥ 10.0). This *Z* score uses Body Surface Area and luminal diameter of the coronary artery. Hemodynamic changes in blood flow combined with coronary artery endothelial dysfunction can cause the formation of intraluminal wall-associated thrombi [3]. Depending on the classification, prophylactic anticoagulation and/or antiplatelet therapy is indicated. When partial thrombotic occlusion occurs, close monitoring with additional imaging techniques is warranted. Rarely patients develop a life threatening thrombotic occlusion requiring coronary artery bypass grafting (CABG) [4–6].

We present a unique case of a 5-year-old girl with giant KD associated CAAs, who presented one and a half year after the acute phase with sudden and progressive thrombotic coronary occlusion despite triple anticoagulation treatment and percutaneous coronary intervention (PCI) with repeated alteplase. CABG was required despite being a technically challenging intervention at this very young age.

## Case presentation

A 5-year-old Indian girl presented at our national referral center for KD in the Netherlands. One year and 6 months before, she was diagnosed with KD while living abroad. On the 8th day of fever she was treated once with 2 g/kg IVIG and high dose aspirin. Despite timely treatment with IVIG, the initial echocardiographic imaging showed the development of giant CAAs in the left anterior descending coronary artery (LAD) (luminal diameter 12 mm, *Z* score + 27) and the right coronary artery (RCA) (luminal diameter 9 mm, *Z* score + 17). She was treated with acetylsalicylic acid (3 mg/kg) and warfarin immediately [1]. Blood tests repeatedly confirmed that the International Normalized Ratio (INR) was within an effective therapeutic range (2.0–3.0). Family history did not reveal coagulation disorders or arterial or venous thromboembolic events.

One and a half years after onset of disease a cCTA was performed which confirmed the giant CAAs (as detected by echocardiography), and showed no signs of thrombosis.

**Fig. 1:**
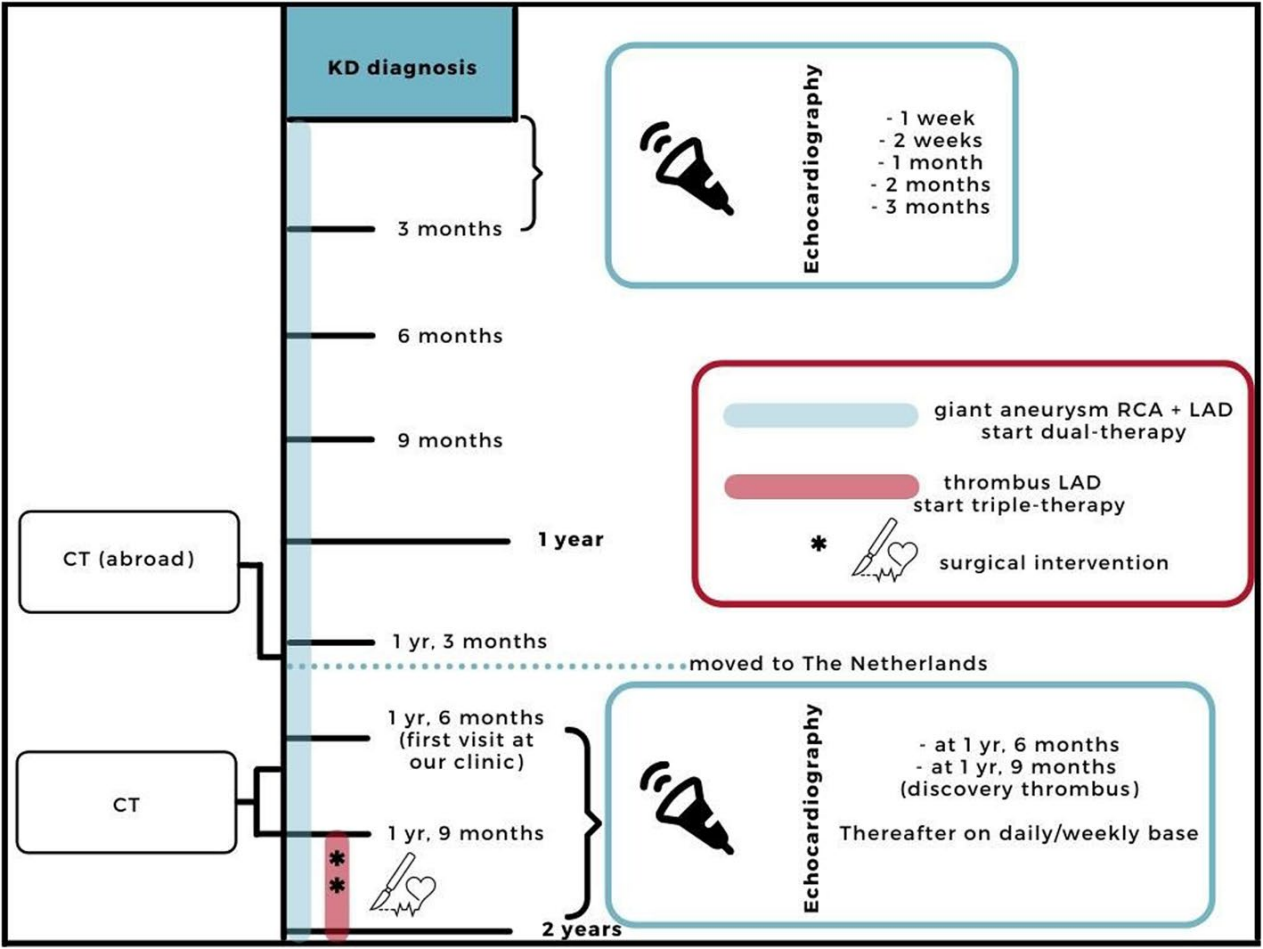
Timeline of the first 2 years after onset of disease

Figure 1 shows a schematic timeline overview of the imaging and intervention performed during the first 2 years of the course of the disease.

Warfarin is not registered in The Netherlands and therefore was replaced by oral acenocoumarol. During this switch the INR was subtherapeutic once (INR 1.7) being again in the therapeutic range within 7 days upon adapting the acenocoumarol dose.

The cCTA scan was repeated upon arrival to the Netherlands shortly after warfarin was replaced by acenocoumarol and showed no signs of thrombosis.

**Fig. 2:**
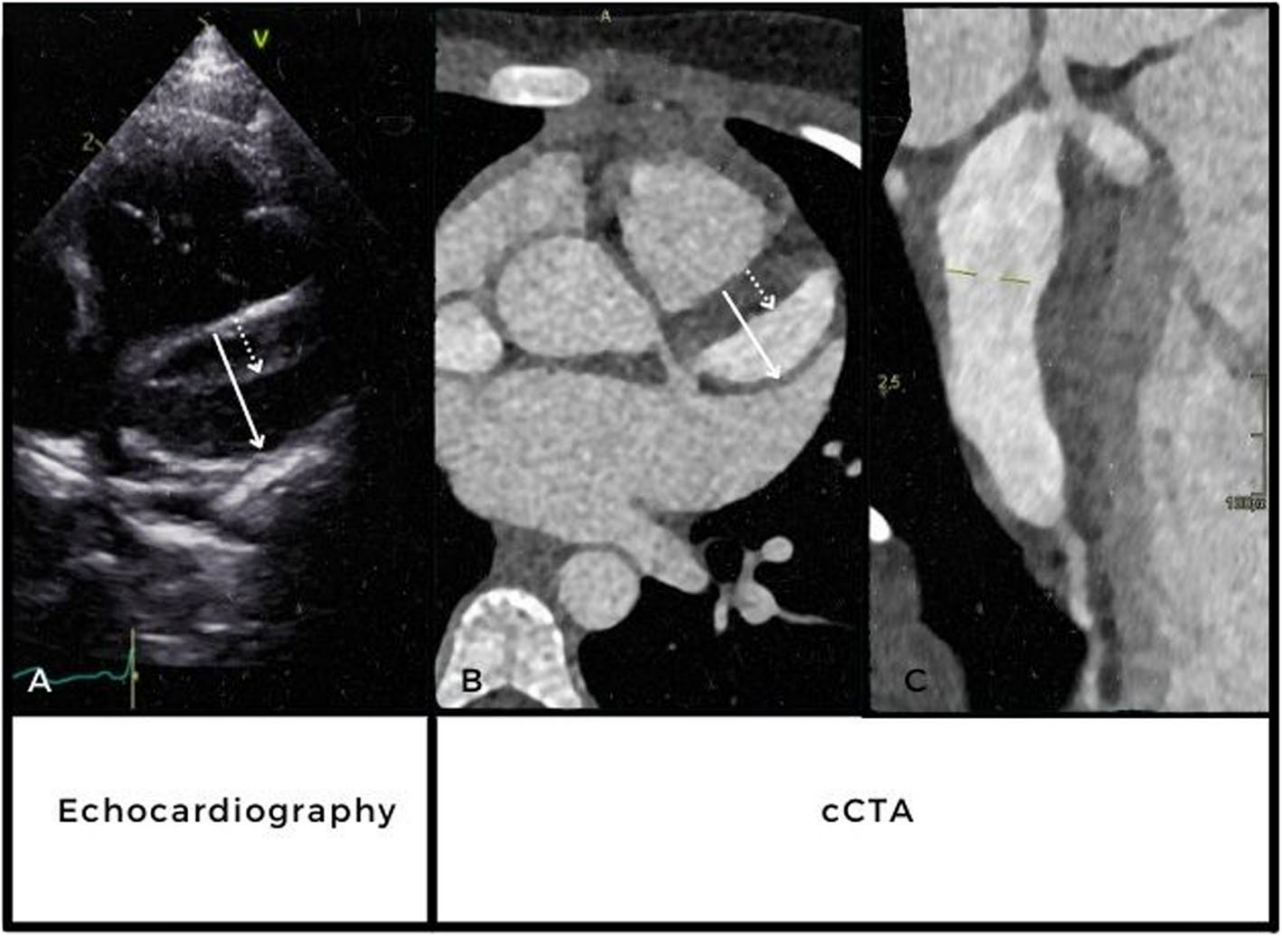
Giant CAA (arrow) in LAD with thrombus (dotted arrow) depicted by echocardiography (**A**) and cCTA (B + C). **C** A reconstruction of the full length of the left main coronary artery. Echocardiography (**A**) and cCTA (**B**) showing thrombus (dotted arrow) in giant CAA with a luminal diameter of 13 mm, equivalent to a *Z* score of 34 (arrow) in LAD

However, three months later, at 1 year and 9 months after the initial onset of disease, a routine echocardiography showed a new echo density in the LAD (Fig. 2A) despite adequate antiplatelet and anticoagulation therapy. A subsequent cCTA confirmed intraluminal thrombus growth causing an a reduction in patent lumen of the dilated LAD (Fig. 2). Clinical symptoms such as angina and/or shortness of breath were absent and the INR was within an effective therapeutic range (2.0–3.0). In order to prevent further thrombus progression, anticoagulation therapy was intensified with the INR targeting at 2.5–3.5, acetylsalicylic acid was increased to 5 mg/kg and clopidogrel 1 mg/kg was added. After a short uneventful clinical observation she was further followed on an outpatient basis.

In spite of the triple anticoagulation therapy, one month later, the patient presented to the Emergency Department with the classical presentation of myocardial ischemia i.e. pain in the left arm, nausea and vomiting. ECG showed a ST-depression and blood test revealed an elevated troponine-T of 0.114 μg/L (normal range 0–0.05μg/L) [7]. Echocardiography showed an end diastolic Left Ventricular Inner Dimension of 40.2 mm and left ventricle ejection fraction of 18.7 44%. Laboratory results showed a consistent increase in NT-proBNP and the ECG showed that the ST-depression progressed into ST-elevation (supplementary Fig. 1). With these findings, the clinical criteria for myocardial ischemia were met [7]. Systemic thrombolysis with continuous infusion of recombinant tissue-type plasminogen activator 0.2–3 mg/kg/hr. (rTPA, alteplase) and continuous nitroglycerine was started. Anticoagulation with acenocoumarol was switched to continuous unfractionated heparin and dual antiplatelet therapy continued with acetylsalicylic acid and prasugrel instead of clopidogrel.

Persistent ST-segment elevation led to a prompt PCI with balloon dilatation with thrombosuction and local thrombolysis with a 15-min drip of rTPA (0.1 mg/kg). Subsequently, 12-h thrombolysis was accomplished following PCI. Blood flow in the LAD restored partially, but a substantial proportion of the thrombus remained detectable.

**Fig. 3:**
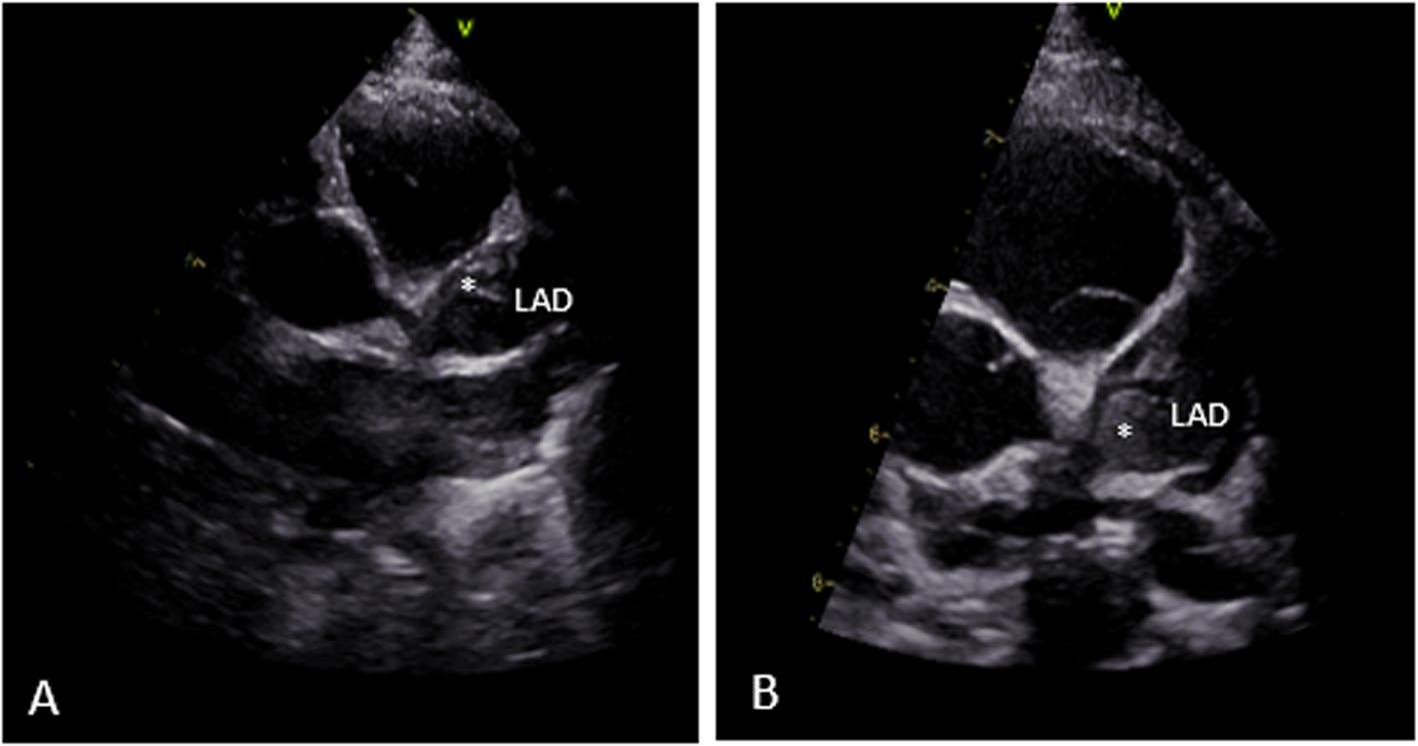
Thrombus growth depicted by echocardiography. **A** Thrombus (*) in LAD. **B** Thrombus (*) growth in the course of 4 days in LAD

**Fig. 4:**
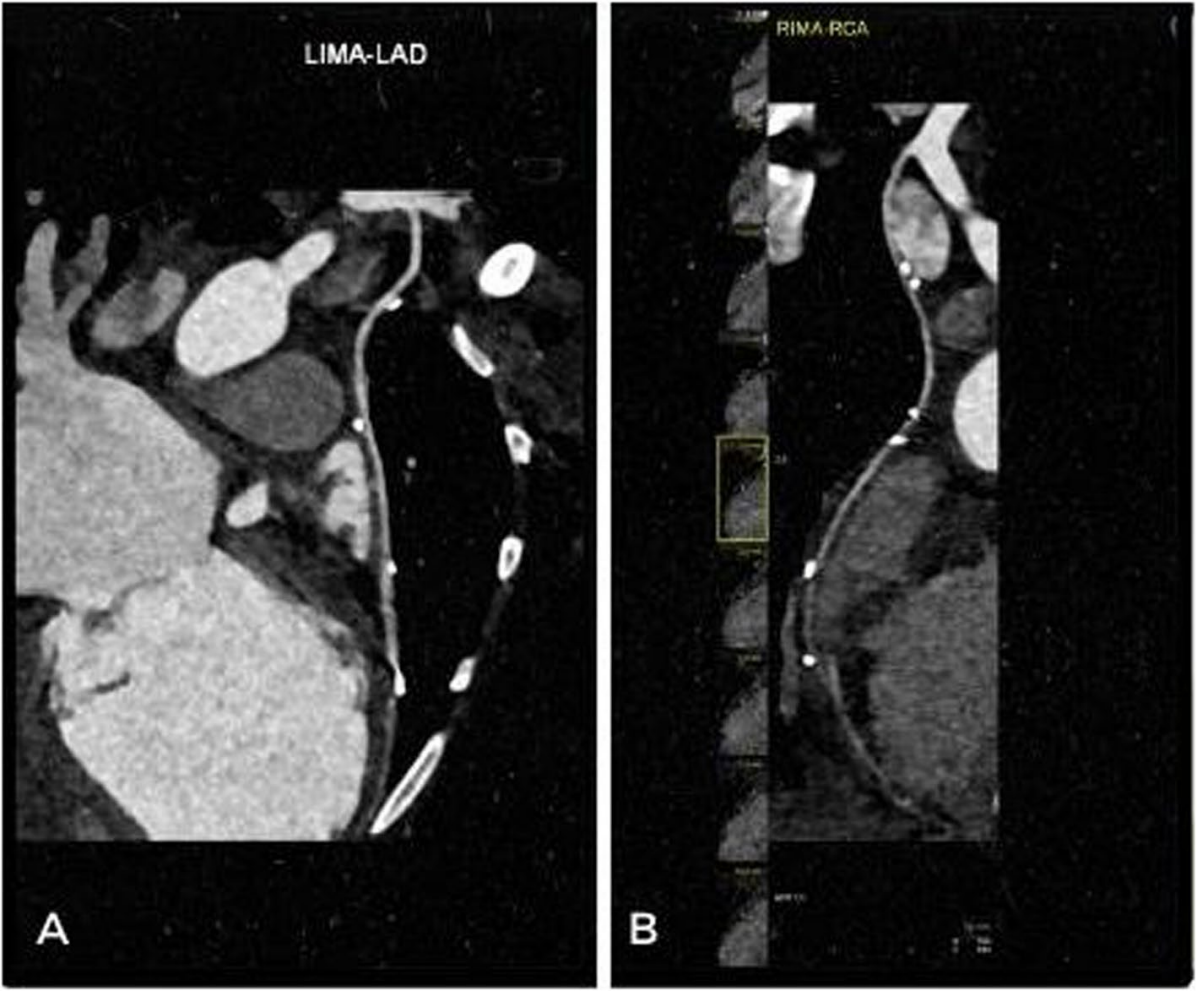
Reconstruction of the LIMA (**A**) and RIMA (**B**) graft by cCTA

The following week gradual improvement in ECG and troponine-T levels was seen and continuous heparin was switched to low molecular weight heparins (nadroparin) with therapeutic antiXa levels. However, after one week echocardiography showed again growth of the thrombus (Fig. 3), for which rTPA and heparin was restarted with reduction of the thrombus. After discontinuation of rTPA which lasted for 6 h, ECG showed yet again an increase in ST-elevation coinciding with a growth of the thrombus by echocardiography. Because of the rapidly progressive growth of the thrombus despite aggressive anticoagulant therapy, CABG was performed. A left internal mammary artery (LIMA) graft to the LAD (Fig. 4A) and a right internal mammary artery (RIMA) graft to the RCA (Fig. 4B) were placed. The thrombus was removed and the CAA in the RCA was clipped.

The now eight-year-old patient is clinically well and leads an active life. The perseverant thrombotic occlusion that caused a major anteroseptal myocardial infarction has led to a lasting compromised cardiac function with a markedly dilated left ventricle and aneurysmatic and abnormal left ventricular wall motion. At 22 months post-CABG, the left ventricular Inner Dimension is 56.9 mm with an ejection fraction of 30%. The patient remains on triple anticoagulation (acenocoumarol, acetylsalicylic acid and prasugrel) without any increased bleeding tendency. She is also treated for heart failure (bisoprolol, enalapril, furosemide, prasugrel and spironolactone) with which the NT-proBNP has shown a decrease during the outpatient follow-up (Suppl. Fig. 2). The patient was not placed on statins.

## Discussion and conclusions

A unique KD case is reported with anticoagulation resistant acute giant CAA thrombus more than a year after onset of disease, despite adequate triple anticoagulation therapy. The girl showed progressive clot formation after repeated alteplase therapy, finally causing permanent myocardial damage.

Latest insights show that mainly giant CAAs (especially with a Z score ≥ 20) are related to clinical complications and major adverse cardiac events because of luminal narrowing, obstructing coronary artery thrombosis, or life-threatening arrhythmias due to ischemia [8]. Therefore, not only correct classification of the size of CAA in the acute phase, but also long-term cardiovascular follow-up is of great importance.

Treatment failure with clopidogrel has been reported in the adult population due to reduced pharmacological effect [9, 10]. To be certain, clopidogrel was substituted by prasugrel because of potential clopidogrel resistance [9, 10]. A CYP2C19 polymorphism (Cyp2C19*1/*2) was found, which is to some extent associated with decreased exposure to the active metabolite of clopidogrel, and results in a delayed stabilization. However, the lower pharmacological effect of clopidogrel cannot explain the progressive nature of the clotting in our patient. Even after optimal dual antiplatelet therapy demonstrated with platelet aggregometry and high intensity anticoagulation, the thrombus progressed. Therefore, this case shows that even triple anticoagulant therapy cannot always prevent ongoing thrombus formation in a giant LAD aneurysm with decreased blood flow and endothelial dysfunction. Therefore, CABG was the only residual option to attempt to restore the coronary circulation. The thrombus in the RCA was removed and the CAA was clipped. Complete thrombus removal in the LAD would have been technically challenging and a high chance of re-thrombosis was expected as the thrombus occurred despite aggressive anticoagulation. The outcome of the chosen management was unfavorable because of myocardial infarction. However, rescue CABG was considered to be the only chance of improving the coronary flow at that moment. Nonetheless, during the follow-up in the outpatient clinic the patient showed clinical improvement and a stable decline in NT-ProBNP (supplementary Fig. 2).

Our case is one of the few very young patients who underwent bilateral IMA grafts. In KD, saphenous vein grafts were initially used in CABG, however, due to the declining patency, especially in children < 10 years of age [11, 12], a living arterial graft (internal mammary artery [IMA]) with the potency of growth was introduced. The first successful use of a single IMA graft in KD patients (at the age of 6 and 10) has been described in 1985 [11]. The first bilateral IMA graft (in an 8 year old patient) was described in 1988 [13]. I Although majority of literature focusses on single IMA grafts in older age [5, 6] 8 patients were reported (with a mean age of 8 years) [14], and 3 very young KD patients (at the age of 3, 4 and 5 years) receiving bilateral IMA grafts [4, 14].

The follow-up of the graft patency in our patient has been evaluated by cCTA and has shown contrast filling into the coronary arteries, confirming a good patency. Long-term follow-up of adolescent patients receiving LIMA or RIMA grafts is very good, ranging from 96 and 84% at 10 years, respectively [15]. Overall patency for internal thoracic artery grafts was 87% at 20 years [6], with only 3 patients at the age of 3, 4, and 6 years are reported with a maximum follow-up of 1, 7 and 8 years [4].

Immediate stenting should always be considered, but was not an option in the presence of the rapidly progressing thrombus formation despite maximal anticoagulation therapy. The decision to perform CABG was made after thorough multidisciplinary consideration, to opt to improve cardiac circulation.

From this case report we can conclude that if a patient shows rapid growth of a thrombus despite triple anticoagulation therapy, due to a state of low-flow in the aneurysm, CABG should be considered very early on to avoid complete occlusion.

## Supplementary Information

**Additional file 1: Supplementary Fig. 1.** In the course of 6 h changes in the ECG were notable indicating myocardial ischemia. A. ST-elevation B**.** ST-depression.

**Additional file 2: Supplementary Fig. 2.** NT-proBNP over the course of 1 year after CABG measured during outpatient follow-up.

## Data Availability

Not applicable.

## Abbreviations

CAA: Coronary artery aneurysm
CABG: Coronary artery bypass grafting
cCTA: Coronary computed tomographic angiography
IMA: Internal mammary artery
INR: International Normalized Ratio
IVIG: Intravenous immunoglobulin
KD: Kawasaki disease
LAD: Left anterior descending artery
LIMA: Left internal mammary artery graft
PCI: Percutaneous coronary intervention
RCA: Right coronary artery
RIMA: Right internal mammary artery
