# Supplementary figures and images for: Myocardial infarction due to thrombotic occlusion despite anticoagulation in Kawasaki disease – a case report
Source: BMC Pediatr. 2022 Feb 12;22:85. doi: 10.1186/s12887-022-03151-2 (PMC8840548; doi:10.1186/s12887-022-03151-2)

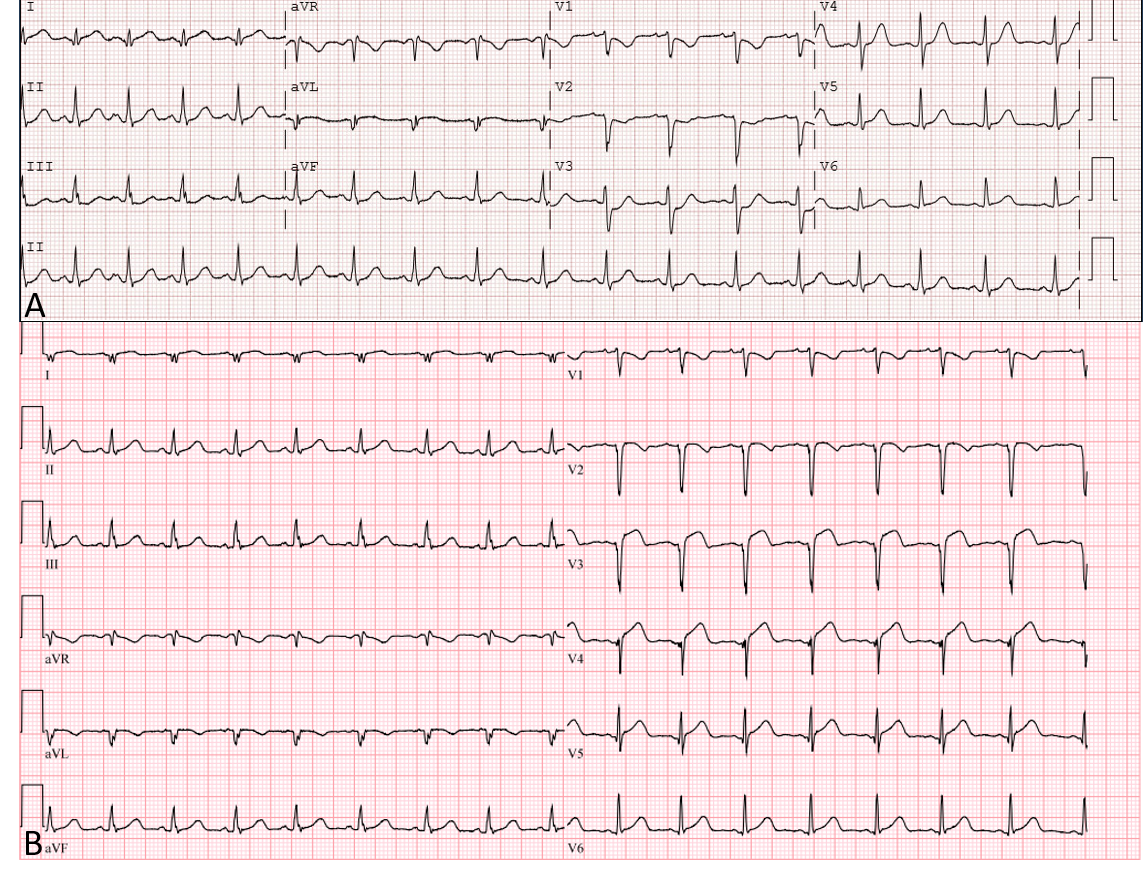

Supplement: Supplementary file 1 — Additional file 1: Supplementary Fig. 1. In the course of 6 h changes in the ECG were notable indicating myocardial ischemia. A. ST-elevation B. ST-depression. [file 12887_2022_3151_MOESM1_ESM.png]

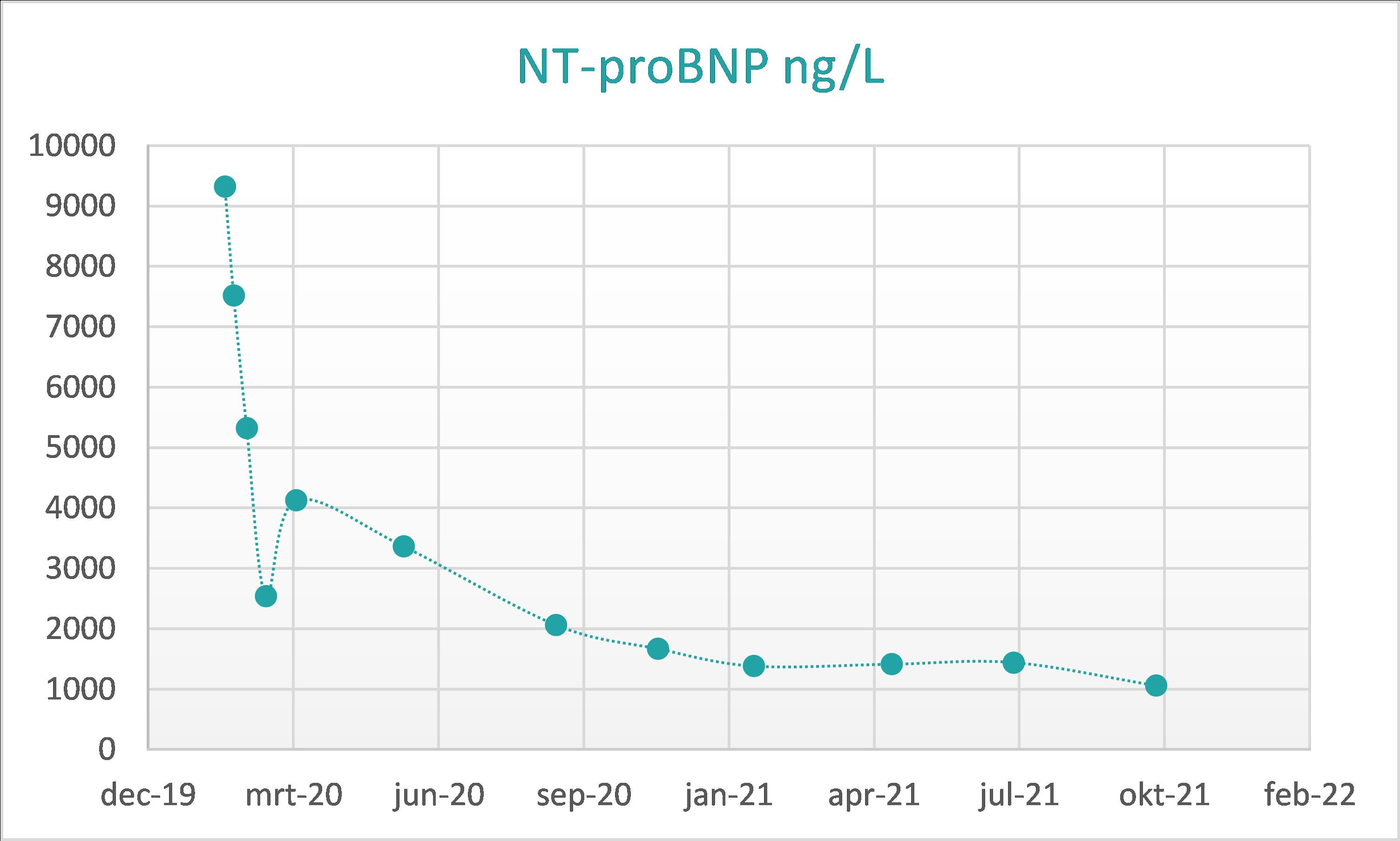

Supplement: Supplementary file 2 — Additional file 2: Supplementary Fig. 2. NT-proBNP over the course of 1 year after CABG measured during outpatient follow-up. [file 12887_2022_3151_MOESM2_ESM.jpg]
